# Influence of dexmedetomidine on postoperative cognitive dysfunction in the elderly: A meta‐analysis of randomized controlled trials

**DOI:** 10.1002/brb3.2665

**Published:** 2022-07-10

**Authors:** Hui Yu, Hui Kang, Jingxiu Fan, Ge Cao, Bin Liu

**Affiliations:** ^1^ Department of Cardiovascular Surgery West China Hospital of Sichuan University Chengdu China; ^2^ Department of Anesthesiology West China Hospital of Sichuan University Chengdu China

**Keywords:** dexmedetomidine, elderly, meta‐analysis, postoperative cognitive dysfunction, surgery

## Abstract

**Introduction:**

Dexmedetomidine (Dex) is suggested to be neuroprotective. However, influence of Dex on postoperative cognitive dysfunction (POCD) in the elderly remains unknown.

**Methods:**

We performed a meta‐analysis of randomized controlled trials (RCTs) to evaluate the effect of Dex on POCD. Relevant studies were obtained by search of PubMed, Embase, and Cochrane's Library databases. A random‐effect model was used to pool the results.

**Results:**

Fourteen RCTs including 1626 adults of 60 years or older who received surgery with general anesthesia were included. Because methodologically diverse scales were used for POCD, eight RCTs with POCD diagnosed with Mini‐Mental State Examination (MMSE) were included in the meta‐analysis, while the remaining six RCTs with POCD diagnosed with other scales were qualitative synthesized. Pooled results of RCTs with MMSE showed that Dex significantly reduced the incidence of POCD (risk ratio: 0.47, 95% confidence interval: 0.37–0.60, *p* < 0.001) with no significant heterogeneity (*I* ^2^ = 0%) or publication bias (*p* for Egger's regression test = 0.579). For the remaining six RCTs with POCD diagnosed with other scales, three of them showed that Dex was associated with a significantly lower incidence of POCD, while the other three RCTs did not show a significant difference.

**Conclusions:**

Dex is associated with a reduced risk of POCD in elderly patients receiving surgeries with general anesthesia, and the results were mainly obtained in studies with POCD diagnosed with MMSE. Based on these findings, Dex may be considered as a preventative measure for POCD in elderly patients.

## Graphical Abstract

A systematic review and meta‐analysis of 14 randomized controlled trials showed that dexmedetomidine is associated with a reduced risk of postoperative cognitive dysfunction in elderly patients receiving surgeries with general anesthesia, and the results were mainly obtained in studies with postoperative cognitive dysfunction diagnosed with MMSE. Based on these findings, Dex may be considered as a preventative measure for postoperative cognitive dysfunction in elderly patients.

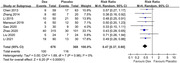

## 1 INTRODUCTION

Postoperative cognitive dysfunction (POCD), which is defined as a decline in cognitive function after the surgery, is a common postoperative cognitive disorder in patients following surgeries with general anesthesia, particularly in the elderly population (Belrose & Noppens, 2019; Brodier & Cibelli, 2021; Urits et al., 2019). Previous studies showed that the incidence of POCD varied between 20% and 40% in people aged 60 years or older (Lin et al., 2020). Moreover, POCD has been related to prolonged hospitalization, impaired functional ability, and increased mortality of patients after surgeries (L. Gao et al., 2005; Ruggiero et al., 2017). Therefore, development of novel strategy to prevent the incidence of POCD is of great clinical significance, especially in the elderly population.

Dexmedetomidine (Dex) is a well‐applied perioperative medication for patients that received surgeries with general anesthesia (Abowali et al., 2021). Pharmacologically, Dex is a highly selective α2‐adrenoreceptor agonist, which exerts various clinical efficacies during perioperative periods, such as sedation, analgesia, anti‐anxiety, and diuresis (Keating, 2015; Lee, 2019). Besides, accumulating evidence showed that Dex may also confer neuroprotective effects (Jiang et al., 2017; Y. Wang et al., 2016). However, previous clinical studies evaluating the influence of Dex on POCD in elderly patients who received surgery showed inconsistent results (J. Chen et al., 2013; Ding et al., 2015; Y. Gao et al., 2020; Y. Li et al., 2015; Z. Li et al., 2021, 2020; Mansouri et al., 2019; Mohamed & Shaaban, 2014; Shi et al., 2020; K. Wang et al., 2015; Xu et al., 2017; Zhang et al., 2014; Zhao et al., 2020; M. Zhou et al., 2019). Some randomized controlled trials (RCTs) suggested that Dex may reduce POCD in the elderly population (Y. Li et al., 2015; Z. Li et al., 2021; Mohamed & Shaaban, 2014; Shi et al., 2020; Zhang et al., 2014; Zhao et al., 2020), while others did not (J. Chen et al., 2013; Ding et al., 2015; Y. Gao et al., 2020; Liu et al., 2020; Mansouri et al., 2019; K. Wang et al., 2015; Xu et al., 2017; M. Zhou et al., 2019). Although two early meta‐analyses (Man et al., 2015; C. Zhou et al., 2016) showed that Dex may be associated with preserved postoperative cognitive function, only RCTs published before 2015 were included. Moreover, the relatively small number of available RCTs prevented further analyses regarding the influences of study characteristics on the outcome (Man et al., 2015; C. Zhou et al., 2016). With the accumulated studies in recent years (Y. Gao et al., 2020; Z. Li et al., 2021; Liu et al., 2020; Mansouri et al., 2019; Shi et al., 2020; Xu et al., 2017; Zhao et al., 2020; M. Zhou et al., 2019), we performed an updated meta‐analysis to evaluate the influence of Dex on the incidence of POCD in the elderly population. Moreover, possible influences of characteristics, such as type of the surgery, regimen of anesthetics, using of Dex loading dose, instrument for POCD measuring, and so on, on the outcome were also studied.

## 2 METHODS

The PRISMA 2020 (Preferred Reporting Items for Systematic Reviews and Meta‐Analyses 2020) statement (Page, McKenzie, et al., 2021a; Page, Moher, et al., 2021b) and the Cochrane Handbook guidelines (Higgins et al., 2021) were followed during the designing and implementation of the study.

### 2.1 Search strategy

PubMed, Embase, and the Cochrane Library (Cochrane Center Register of Controlled Trials) databases were searched for relevant studies with a combined strategy of: (1) “dexmedetomidine”; (2) “cognition” OR “cognitive” OR “dementia” OR “cognit*” OR “deliri*” OR “mild cognitive impairment*” OR “mild‐cognitive impairment*” OR “neuropsycholo*” OR “POCD” OR “postoperative cognitive” OR “post‐operative cognitive” OR “MMSE” OR “mini‐mental state examination” OR “cerebral function” OR “neurocognit*” OR “encephalopath” OR “cognition” OR “cognitive” OR “delirium”; and (3) “random” OR “randomized” OR “randomised” OR “randomly” OR “allocated” OR “control” OR “placebo.” Only clinical studies were considered. The references of related reviews and original articles were also searched as a complementation. The final database search was conducted on June 20, 2021.

### 2.2 Study selection

Studies that fulfilled the following criteria were included: (1) Articles published in English or Chinese; (2) designed as parallel‐group RCTs; (3) included elderly patients (60 years or older) scheduled for surgery with general anesthesia who were randomly allocated to a Dex treatment group or a control group with placebo or blank treatment; and (4) reported the incidence of POCD in the perioperative periods. The diagnostic criteria of POCD outcomes in the meta‐analysis were in accordance with that applied in the included studies. Reviews, studies with non‐elderly patients, preclinical studies, observational studies, and repeated reports were excluded.

### 2.3 Data extraction and quality assessment

Database search, data extraction, and quality evaluation were conducted by two independent authors. If disagreement occurred, it was resolved by discussion with the corresponding author. We extracted data regarding study information (first author, publication year, and study country), study design (blind or open‐label), patient information (number of participants, range of age, and sex), surgery type, perioperative anesthetics and anesthesia depth monitoring, regimens of Dex and control, and diagnostic strategy for POCD. Quality evaluation was achieved using the Cochrane's Risk of Bias Tool (Higgins et al., 2021) according to the following aspects: (1) Random sequence generation; (2) allocation concealment; (3) blinding of participants and personnel; (4) blinding of outcome assessors; (5) incomplete outcome data; (6) selective outcome reporting; and (7) other potential bias.

### 2.4 Statistical analysis

Incidence of POCD was separately evaluated via risk ratios (RRs) and their 95% confidence intervals (CIs) in this meta‐analysis. We used the Cochrane's Q test to detect the heterogeneity (Higgins et al., 2021). The *I* ^2^ statistic was also calculated, and an *I* ^2^ > 50% reflected significant heterogeneity. Pooled analyses were calculated using a random‐effect model because this method incorporates the influence of potential heterogeneity and retrieves a more generalized result (Higgins et al., 2021). Sensitivity analysis by excluding one study at a time was used to evaluate the influence of each study on the pooled results of the meta‐analysis (Higgins et al., 2021). Predefined subgroup analyses were used to evaluate the possible influences of study characteristics on the effect of Dex on POCD risk. Publication bias was evaluated by visual inspection of funnel plots, and the Egger's regression asymmetry test (Higgins et al., 2021). *p* values <0.05 were considered statistically significant. The RevMan (Version 5.1; Cochrane, Oxford, UK) and Stata software (Version 12.0; Stata, College Station, TX) were applied for statistical analyses.

## 3 RESULTS

### 3.1 Search results

**FIGURE 1:**
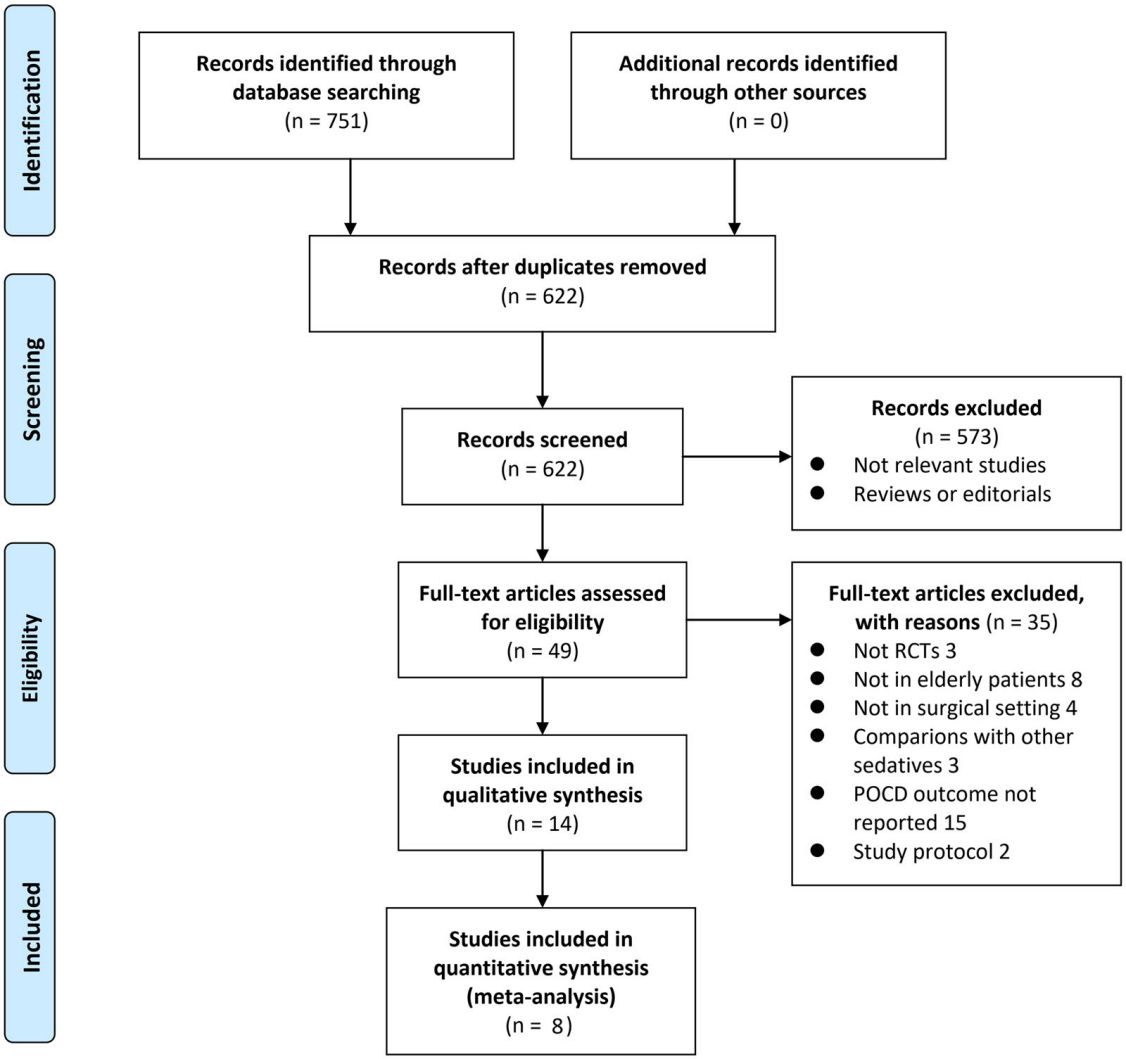
Flow chart of literature search

The process of database search and study identification is shown in Figure 1. Briefly, 751 articles were obtained through the database search, and 622 were retrieved after exclusion of duplicated records. Among them, 573 articles were subsequently excluded based on titles and abstracts primarily because these studies were irrelevant to the aim of the meta‐analysis. Of the 49 articles that underwent full‐text review, 35 were further excluded for the reasons presented in Figure 1. Finally, 14 RCTs (J. Chen et al., 2013; Ding et al., 2015; Y. Gao et al., 2020; Y. Li et al., 2015; Z. Li et al., 2021; Liu et al., 2020; Mansouri et al., 2019; Mohamed & Shaaban, 2014; Shi et al., 2020; K. Wang et al., 2015; Xu et al., 2017; Zhang et al., 2014; Zhao et al., 2020; M. Zhou et al., 2019) were included.

### 3.2 Study characteristics

Because methodologically diverse scales were used for POCD, eight RCTs with POCD diagnosed with the Mini‐Mental State Examination (MMSE) were included in the meta‐analysis (J. Chen et al., 2013; Y. Gao et al., 2020; Y. Li et al., 2015; Z. Li et al., 2021; Liu et al., 2020; Mansouri et al., 2019; Zhang et al., 2014; Zhao et al., 2020), while the remaining six RCTs with POCD diagnosed with other scales were qualitative synthesized (Ding et al., 2015; Mohamed & Shaaban, 2014; Shi et al., 2020; K. Wang et al., 2015; Xu et al., 2017; M. Zhou et al., 2019). Table 1 shows the characteristics of the included studies. Overall, 14 RCTs with 1626 elderly patients were included in the current meta‐analysis (J. Chen et al., 2013; Ding et al., 2015; Y. Gao et al., 2020; Y. Li et al., 2015; Z. Li et al., 2021; Liu et al., 2020; Mansouri et al., 2019; Mohamed & Shaaban, 2014; Shi et al., 2020; K. Wang et al., 2015; Xu et al., 2017; Zhang et al., 2014; Zhao et al., 2020; M. Zhou et al., 2019). Twelve of the studies were performed in China (J. Chen et al., 2013; Ding et al., 2015; Y. Gao et al., 2020; Y. Li et al., 2015; Z. Li et al., 2021; Liu et al., 2020; Shi et al., 2020; K. Wang et al., 2015; Xu et al., 2017; Zhang et al., 2014; Zhao et al., 2020; M. Zhou et al., 2019), and the other two were performed in Egypt and Iran (Mansouri et al., 2019; Mohamed & Shaaban, 2014), respectively. All of these studies were double‐blinded RCTs except for two studies, which were single‐blinded (Y. Gao et al., 2020; Zhang et al., 2014). For most of the included studies, non‐cardiac surgeries were performed (J. Chen et al., 2013; Ding et al., 2015; Y. Li et al., 2015; Z. Li et al., 2021; Liu et al., 2020; Mansouri et al., 2019; Mohamed & Shaaban, 2014; Shi et al., 2020; K. Wang et al., 2015; Xu et al., 2017; Zhang et al., 2014; Zhao et al., 2020), while for two studies, cardiac surgeries were performed (Y. Gao et al., 2020; M. Zhou et al., 2019). For three studies (Ding et al., 2015; Mohamed & Shaaban, 2014; M. Zhou et al., 2019), sevoflurane was used during anesthesia with intravenous anesthetics such as propofol, remifentanil, or sufentanil. A loading dose and subsequent continuously intravenous administration of Dex was applied in most of the included studies except for two studies (Shi et al., 2020; K. Wang et al., 2015), in which a loading dose of Dex was not applied. Dex was administered during surgical procedure in all of the included studies, while in two studies, Dex was also administered in intensive care unit (ICU) after the surgery (K. Wang et al., 2015; Zhao et al., 2020). Placebo of normal saline was applied in all the included studies. As for the evaluation strategy of POCD, the MMSE was performed in eight studies (J. Chen et al., 2013; Y. Gao et al., 2020; Y. Li et al., 2015; Z. Li et al., 2021; Liu et al., 2020; Mansouri et al., 2019; Zhang et al., 2014; Zhao et al., 2020), while in the other studies, instruments such as the Montreal Cognitive Assessment (MoCA), Stroop color test, and Chinese Neurocognitive Scale were used (Ding et al., 2015; Mohamed & Shaaban, 2014; Shi et al., 2020; K. Wang et al., 2015; Xu et al., 2017; M. Zhou et al., 2019). Patients with POCD were identified within 24 h after surgery in all of the included studies.

**TABLE 1:** Characteristics of the included RCTs

Study	Country	Design	Surgical procedure	No. of patients	Age range (years)	Male (%)	Anesthesia regimen	Anesthesia depth monitoring	Dex regimens	Loading dose of Dex	Timing and duration of Dex	Control	Diagnosis of outcomes
POCD diagnosed with MMSE													
Chen 2013	China	R, DB, PC	Laparoscopic cholecystectomy	122	60∼75	52.5	Propofol and remifentanil	NR	1 ug/kg for 10 min after induction, and 0.4 ug/kg/h until the end of surgery	Yes	During surgery	NS	Postoperative MMSE < 27
Zhang 2014	China	R, SB, PC	Laparoscopic surgery for colorectal cancer	80	65∼85	58.8	Propofol and remifentanil	Nareotrend Index 45∼55	0.5 ug/kg for 15 min before induction, and 0.2, 0.5, or 0.8 ug/kg/h until the end of surgery	Yes	During surgery	NS	Postoperative MMSE decline ≥ 2
Li 2015	China	R, DB, PC	Laparoscopic cholecystectomy	100	60∼75	54	Propofol and remifentanil	BIS 40∼60	1 ug/kg for 10 min after induction, and 0.4 ug/kg/h until the end of surgery	Yes	During surgery	NS	A postoperative decrease of minimally 1 SD in MMSE
Mansouri 2019	Iran	R, DB, PC	Cataract surgery	100	≥ 65	44	Nitrous oxide	NR	1 ug/kg for 10 min at induction	Yes	During surgery	NS	Postoperative MMSE < 26
Gao 2020	China	R, SB, PC	Minimally invasive CABG	60	65∼75	48.3	Propofol and sufentanil	BIS 40∼60	1 ug/kg for 15 min after induction, and 0.3–0.5 ug/kg/h until the end of surgery	Yes	During surgery	NS	Postoperative decline of MMSE
Zhao 2020	China	R, DB, PC	Non‐cardiac surgery	416	≥ 65	56.3	Propofol and remifentanil	BIS 40∼60	1 ug/kg for 15 min before induction, and 100, 200, or 400 ug in patient‐controlled intravenous analgesia	Yes	During surgery and in ICU	NS	Postoperative MMSE decline ≥ 2
Liu 2020	China	R, DB, PC	Colorectal cancer radical resection	48	≥ 65	58.3	Propofol and remifentanil	BIS 40∼55	0.5 μg/kg for 15 min at induction and 0.6 μg/kg/h from induction to the end of surgery	Yes	During surgery	NS	Postoperative MMSE decline ≥ 2
Li 2021	China	R, DB, PC	Spine surgery	120	65∼90	56.7	Propofol and remifentanil	BIS 40∼60	0.3 ug/kg for 10 min before induction, and 0.2, 0.5, and 0.8 ug/kg/h until the end of surgery	Yes	During surgery	NS	A decrease of minimally 1 SD in 2 or more postoperative neurocognitive tests by MMSE
POCD diagnosed with MoCA													
Xu 2017	China	R, DB, PC	Laparoscopic ovarian cystectomy	96	63∼85	0	Propofol and sufentanil	BIS 45∼55	0.8 ug/kg/h for 10 min before induction, and 0.5 ug/kg/h until the end of surgery	Yes	During surgery	NS	Postoperative MoCA < 27 or postoperative MoCA decline ≥ 2
Zhou 2019	China	R, DB, PC	Cardiac surgery	76	60∼80	46.1	Sevoflurane, propofol and sufentanil	BIS 40∼60	0.4 ug/kg/h until the end of surgery	No	During surgery	NS	A decrease of minimally 1 SD in 2 or more postoperative neurocognitive tests by MoCA
POCD diagnosed with the Chinese Neurocognitive Scale													
Wang 2015	China	R, DB, PC	Spine surgery	152	≥ 60	50.7	Propofol and remifentanil	BIS 50∼60	3 ug/kg in patient‐controlled intravenous analgesia	No	During surgery and in ICU	NS	A decrease of minimally 1 SD in 2 or more postoperative neurocognitive tests by Chinese Neurocognitive Scale
Ding 2015	China	R, DB, PC	Robot‐assisted laparoscopic radical prostatectomy	100	65∼80	100	Sevoflurane, propofol and remifentanil	BIS 40∼60	0.8 ug/kg/h for 10 min before induction, and 0.3 ug/kg/h until the end of surgery	Yes	During surgery	NS	A decrease of minimally 1 SD in 2 or more postoperative neurocognitive tests by Chinese Neurocognitive Scale
POCD diagnosed with other scales													
Mohamed 2014	Egypt	R, DB, PC	Abdominal surgery	50	≥ 60	90	Sevoflurane, fentanyl	NR	1 ug/kg/h for 10 min before induction, and 0.4 ug/kg/h until the end of surgery	Yes	During surgery	NS	A decrease of minimally 1 SD in 2 or more postoperative neurocognitive tests by Stroop color test
Shi 2020	China	R, DB, PC	Thoracoscopic lobectomy	106	≥ 65	100	Propofol, remifentanil and cisatracurium	BIS 45∼60	0.5 μg/kg/h from induction to the end of surgery	No	During surgery	NS	A decrease of minimally 1 SD in 2 or more postoperative neurocognitive tests by a comprehensive test scale of four domains

Abbreviations: RCT, randomized controlled trials; DB, double blind; SB, single blind; PC, placebo controlled; CABG, coronary artery bypass grafting; NR, not reported; BIS, Bispectral index; Dex, Dexmedetomidine; ICU, intensive care unit; NS, normal saline; MMSE, Mini‐Mental State Examination; SD, standard deviation; MoCA, Montreal Cognitive Assessment; POCD, postoperative cognitive dysfunction.

### 3.3 Data quality

Table 2 shows the details of study quality evaluation. All of these studies were double‐blinded RCTs except for two studies, which were single‐blinded (Y. Gao et al., 2020; Zhang et al., 2014). Methods of random sequence generation were reported in nine RCTs (Ding et al., 2015; Y. Li et al., 2015; Z. Li et al., 2021; Liu et al., 2020; Mohamed & Shaaban, 2014; Shi et al., 2020; K. Wang et al., 2015; Zhao et al., 2020; M. Zhou et al., 2019), and information of allocation concealment was reported in five RCTs (J. Chen et al., 2013; Z. Li et al., 2021; Liu et al., 2020; Mohamed & Shaaban, 2014; M. Zhou et al., 2019). Incomplete outcome data, selective reporting, and other sources of biases were judged to be of low risks in all of the included studies.

**TABLE 2:** Details of quality evaluation of the included RCTs via the Cochrane's Risk of Bias Tool

Study	Random sequence generation	Allocation concealment	Blinding of participants	Blinding of outcome assessment	Incomplete outcome data addressed	Selective reporting	Other sources of bias
POCD diagnosed with MMSE							
Chen 2013	Unclear	Low	Low	Low	Low	Low	Low
Zhang 2014	Unclear	Unclear	Low	Unclear	Low	Low	Low
Li 2015	Low	Unclear	Low	Low	Low	Low	Low
Mansouri 2019	Unclear	Unclear	Low	Low	Low	Low	Low
Gao 2020	Unclear	Unclear	Low	Unclear	Low	Low	Low
Zhao 2020	Low	Unclear	Low	Low	Low	Low	Low
Liu 2020	Low	Low	Low	Low	Low	Low	Low
Li 2021	Low	Low	Low	Low	Low	Low	Low
POCD diagnosed with MoCA							
Xu 2017	Unclear	Unclear	Low	Low	Low	Low	Low
Zhou 2019	Low	Low	Low	Low	Low	Low	Low
POCD diagnosed with the Chinese Neurocognitive Scale							
Wang 2015	Low	Unclear	Low	Low	Low	Low	Low
Ding 2015	Low	Unclear	Low	Low	Low	Low	Low
POCD diagnosed with other scales							
Mohamed 2014	Low	Low	Low	Low	Low	Low	Low
Shi 2020	Low	Unclear	Low	Low	Low	Low	Low

Abbreviations: RCTs, randomized controlled trials; MMSE, Mini‐Mental State Examination; MoCA, Montreal Cognitive Assessment; POCD, postoperative cognitive dysfunction.

### 3.4 Meta‐analysis for the studies of POCD evaluated using the MMSE

**FIGURE 2:**
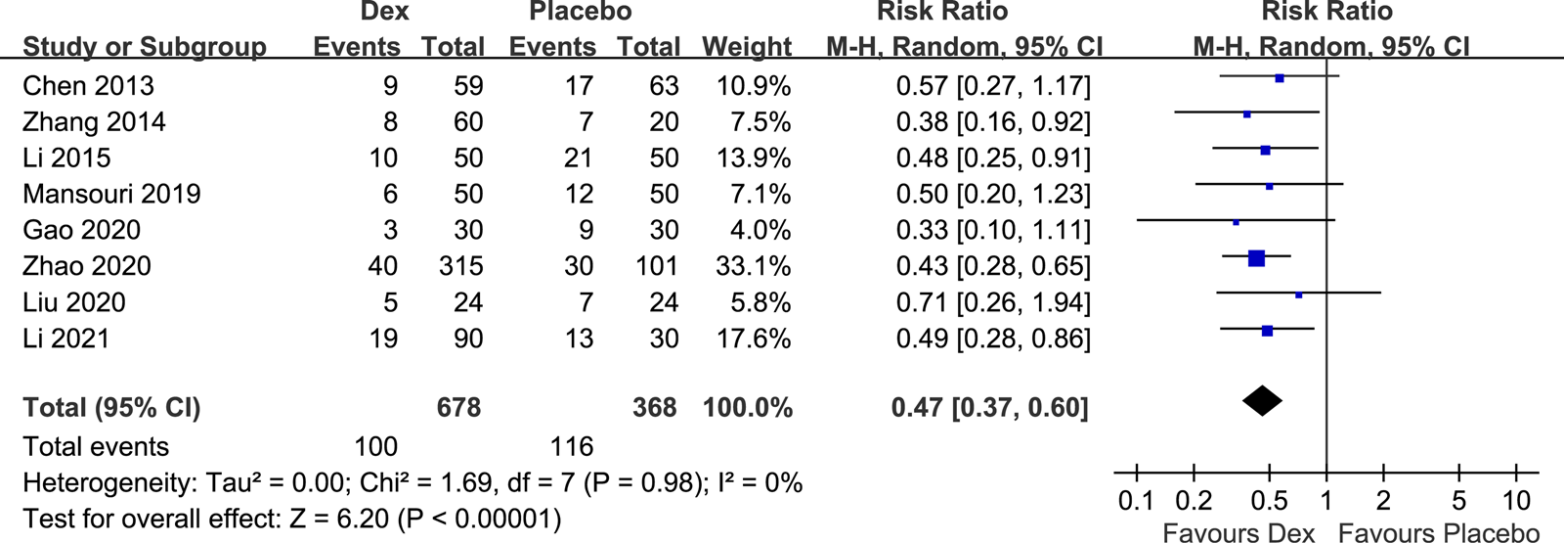
Forest plots for the meta‐analysis of effect of Dex on the risk of POCD in elderly population in studies of POCD diagnosed with the MMSE

Pooled results of eight RCTs (J. Chen et al., 2013; Y. Gao et al., 2020; Y. Li et al., 2015; Z. Li et al., 2021; Liu et al., 2020; Mansouri et al., 2019; Zhang et al., 2014; Zhao et al., 2020) with POCD evaluated using the MMSE showed that Dex significantly reduced the incidence of POCD (RR: 0.47, 95% CI: 0.37–0.60, *p* < 0.001) in elderly patients with no significant heterogeneity (*p* for Cochrane's Q test = 0.98, I^2^ = 0%; Figure 2). All of these eight RCTs did not include sevoflurane in the anesthesia regimen, and Dex was all administered with a loading dose. Sensitivity analysis by excluding one RCT at a time showed consistent results (RR: 0.46 to 0.49, *p* values all < 0.05). Subgroup analysis showed consistent results in single‐ and double‐blinded studies (*p* for subgroup analysis = 0.46, Table 3). Specifically, sensitivity analyses limited to studies from China (RR: 0.47, 95% CI: 0.360.60, *p* < 0.001), patients with non‐cardiac surgery (RR: 0.47, 95% CI: 0.37–0.61, *p* < 0.001), and studies with Dex use only within the surgery process (RR: 0.47, 95% CI: 0.37–0.59, *p* < 0.001; Table 3) showed consistent results.

**TABLE 3:** Results of subgroup and sensitivity analyses for the meta‐analysis of Dex on POCD evaluated by MMSE

Study characteristics	Datasets number	RR (95% CI)	I^2^	P for subgroup effect	P for subgroup difference
Design					
Double blind	6	0.48 [0.37, 0.62]	0%	< 0.001	
Single blind	2	0.36 [0.18, 0.74]	0%	0.005	0.46
Only Chinese studies	7	0.47 [0.36, 0.60]	0%	< 0.001	
Only non‐cardiac surgeries	7	0.47 [0.37, 0.61]	0%	< 0.001	
Only Dex used in surgery	7	0.47 [0.37, 0.59]	0%	< 0.001	

Abbreviations: RR, risk ratio; CI, confidence interval; Dex, dexmedetomidine.

### 3.5 Qualitative synthesis for the studies of POCD evaluated using other instruments

Results of the remaining six RCTs with POCD diagnosed with instruments other than MMSE are summarized in Table 4. Two Chinese studies used MoCA as the instruments for the detection of POCD in elderly patients after surgeries (Xu et al., 2017; M. Zhou et al., 2019). Although the incidence of POCD seemed lower in patients receiving Dex as compared to those of the control group, the differences were not statistically significant (Xu et al., 2017; M. Zhou et al., 2019). For another two studies of POCD diagnosed with the Chinese Neurocognitive Scale, one study showed that Dex significantly reduced the incidence of POCD as compared to control (K. Wang et al., 2015), while the other study failed to show a significant difference of POCD incidence between patients allocated to the Dex and control groups (Ding et al., 2015). The remaining two studies, using Stroop color test and the comprehensive test scale of four domains for the diagnosis of POCD, respectively, showed that Dex was effective in reducing the incidence of POCD in elderly patients after surgeries (Mohamed & Shaaban, 2014; Shi et al., 2020).

**TABLE 4:** Results of studies with POCD diagnosed with scales other than MMSE

Study	Diagnosis scale for POCD	Incidence of POCD in Dex group	Incidence of POCD in control group	P for difference of POCD incidence
Xu 2017	MoCA	0% (0/48)	4.2% (2/48)	0.29
Zhou 2019	MoCA	15.8% (6/38)	31.6% (12/38)	0.12
Wang 2015	Chinese Neurocognitive Scale	8.0% (6/75)	19.5% (15/77)	0.04
Ding 2015	Chinese Neurocognitive Scale	22% (11/50)	34% (17/50)	0.19
Mohamed 2014	Stroop color test	13.3% (8/60)	35% (7/20)	< 0.001
Shi 2020	A comprehensive test scale of four domains	13.2% (7/53)	35.8% (19/53)	0.01

Abbreviations: RR, risk ratio; CI, confidence interval; Dex, dexmedetomidine; MMSE, Mini‐Mental State Examination; MoCA, Montreal Cognitive Assessment; POCD, postoperative cognitive dysfunction.

### 3.6 Publication bias

**FIGURE 3:**
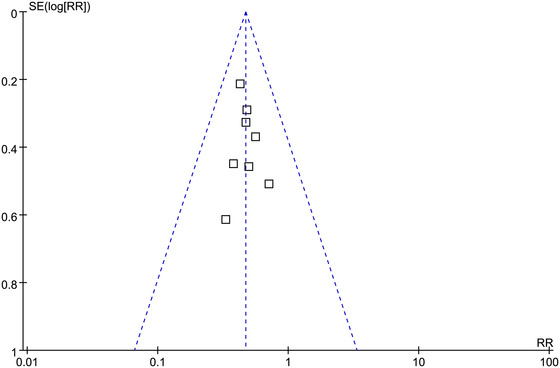
Funnel plots for the publication bias within the meta‐analysis of effect of Dex on the risk of POCD in elderly population in studies of POCD diagnosed with the MMSE

The funnel plots for the meta‐analysis of POCD were symmetrical, suggesting low risk of publication bias (Figure 3). Egger's regression test also showed low risk of publication bias (*p* for Egger's regression test = 0.579).

## 4 DISCUSSION

In this study, by pooling the results of available RCTs, the results of the meta‐analysis showed that compared to placebo, Dex significantly reduced the incidence of POCD as evaluated by MMSE in the elderly population following surgery with general anesthesia. Besides, results of subgroup analyses showed that the potential preventative efficacy of Dex on POCD in elderly population was consistent in single‐blind and double‐blind studies. Sensitivity analyses showed consistent results in studies from China, in patients with noncardiac surgeries, and in studies with Dex administered within the surgeries only. For the remaining six studies with POCD evaluated by instruments other than MMSE, three of them showed Dex could significantly reduce the risk of POCD, while the other three showed a nonsignificantly reduced incidence of POCD following Dex. Taken together, these findings indicated that Dex is associated with a reduced risk of POCD in elderly patients receiving surgeries with general anesthesia, and the results were mainly obtained in studies with POCD diagnosed with MMSE. Based on these findings, Dex may be considered as a preventative measure for POCD in these patients.

To our knowledge, two previous meta‐analyses (Man et al., 2015; C. Zhou et al., 2016) published in 2015 and 2016, respectively, have devalued the influences of Dex on postoperative cognitive function. One study including RCTs published before 2015 showed that perioperative dexmedetomidine treatment is associated with significantly better neurocognitive function postoperatively in comparison with both saline controls and comparator anesthetics (Man et al., 2015). However, this study focused on the changes of MMSE scores after surgery, rather than the incidence of POCD, and included patients with a wide range of ages rather than the elderly population (Man et al., 2015). The other meta‐analysis combined the results of RCTs published until 2015 and showed that dexmedetomidine reduced the incidence of POCD in elderly patients after general anesthesia (C. Zhou et al., 2016). However, results of studies comparing Dex with placebo and other active sedatives were both combined, which made it difficult to interpret the results. Moreover, neither of the meta‐analyses included subgroup analyses to evaluate whether the results were affected by differences of study characteristics. Since considerable RCTs have been published after these meta‐analyses (Man et al., 2015; C. Zhou et al., 2016), we performed an updated analysis to systematically evaluate the influence of Dex on the incidence of POCD in elderly patients after surgery with general anesthesia. Our study has several strengths compared to the previous ones. Firstly, we adopted rigorous literature search, as well as strict inclusion and exclusion criteria to focus on the comparison between Dex and placebo in elderly patients after surgery. Secondly, an up‐to‐date literature search was performed and the numbers of studies and patients included in this meta‐analysis is much larger than the previous ones. Finally, considering the potential clinical heterogeneity which may result due to the difference in instruments used for the diagnosis of POCD, we quantitatively evaluated the influence of Dex on POCD in studies using the MMSE in a meta‐analysis, and qualitatively synthesized the results of the studies using other instruments for the diagnosis of POCD. Overall, we found that perioperative use of Dex is associated with a significantly reduced risk of POCD in elderly patients after general anesthesia. Sensitivity analysis confirmed the robustness of the finding, which was not primarily driven by either of the included studies. Subgroup analyses also showed that the possible preventative efficacy of Dex for POCD was not significantly affected by difference of study design. Taken together, based on these findings, Dex should be recommended as a potential preventative strategy for POCD in elderly patients.

Pathologically, neuroinflammation induced by surgery has been considered as one of the primary mechanisms underlying the pathogenesis of POCD (Luo et al., 2019; Umholtz & Nader, 2017). Accumulating evidence from animal studies showed that Dex could alleviate neuroinflammation induced by surgery or lipopolysaccharide via regulation of systematic inflammatory cytokines including interleukin 1β, tumor necrosis factor‐α, and NF‐κB (N. Chen et al., 2019), inhibiting the expressions of Toll‐like receptor 4 (Yamanaka et al., 2017; X. Y. Zhou et al., 2020), and through α2 adrenoceptor‐mediated anti‐inflammatory pathways (R. Li et al., 2020). However, a recent clinical study showed that Dex preserved postoperative cognitive function in elderly patients who received total knee arthroplasty without significant modulation on peripheral inflammation (Mei et al., 2020), suggesting mechanisms besides anti‐inflammation are also involved. Future studies are warranted to determine the mechanisms underlying the benefits of Dex on postoperative cognitive function in the elderly.

This study also has limitations. Firstly, as previously indicated, instruments for neurocognitive testing and diagnostic criteria for POCD varied among the included studies, and the difference in the definition of POCD may affect the results of the meta‐analysis. In view of the emerged consensus regimens for neurocognitive testing and diagnostic criteria for POCD, such as the Recommendations for the Nomenclature of Cognitive Change associated with Anaesthesia and Surgery (2018) (Evered et al., 2018), studies evaluating the possible preventative strategies for POCD diagnosed with standardized criteria are needed. Furthermore, most of the studies were performed in Chinese population, and all of the studies were performed in the developing countries. Studies from developed countries are needed to validate the consistency of the findings. Accordingly, role of Dex on POCD in elderly population with other ethnicities remains to be evaluated. In addition, we did not have access to the individual patient data. Accordingly, potential influences of patient or study characteristics on the outcomes of the meta‐analysis were unable to be evaluated, such as the sex, comorbidities, and concurrent medications of the patients. Finally, studies are needed to determine whether the differences in the regimens and doses of Dex could affect the possible preventative efficacy of Dex on POCD in the elderly.

## 5 CONCLUSION

In conclusion, results of this meta‐analysis showed that Dex is associated with a reduced risk of POCD in elderly patients receiving surgeries with general anesthesia, and the results were mainly obtained in studies with POCD diagnosed with MMSE. These findings support that Dex should considered as a preventative measure for POCD in this population.

## CONFLICT OF INTEREST

The authors declare that they have no conflicts of interest.

## AUTHOR CONTRIBUTIONS

HY, GC and BL contributed to the conception and design of the study. HY and HK performed literature search, study identification, quality evaluation, and data extraction. HY, HK, and JF performed the statistical analysis. HY, GC, and BL wrote the first draft of the manuscript. All authors contributed to manuscript revision, and read and approved the submitted version.

### PEER REVIEW

The peer review history for this article is available at https://publons.com/publon/10.1002/brb3.2665

## Data Availability

All data generated or analyzed during this study are included in this article. Further enquiries can be directed to the corresponding author.
